# What’s in the Pool? A Comprehensive Identification of Disinfection By-products and Assessment of Mutagenicity of Chlorinated and Brominated Swimming Pool Water

**DOI:** 10.1289/ehp.1001965

**Published:** 2010-09-12

**Authors:** Susan D. Richardson, David M. DeMarini, Manolis Kogevinas, Pilar Fernandez, Esther Marco, Carolina Lourencetti, Clara Ballesté, Dick Heederik, Kees Meliefste, A. Bruce McKague, Ricard Marcos, Laia Font-Ribera, Joan O. Grimalt, Cristina M. Villanueva

**Affiliations:** 1 National Exposure Research Laboratory, U.S. Environmental Protection Agency, Athens, Georgia, USA; 2 National Health and Environmental Effects Research Laboratory, U.S. Environmental Protection Agency, Research Triangle Park, North Carolina, USA; 3 Centre for Research in Environmental Epidemiology, Barcelona, Spain; 4 Municipal Institute of Medical Research, Hospital del Mar, Barcelona, Spain; 5 CIBER Epidemiología y Salud Pública, Barcelona, Spain; 6 Medical School, University of Athens, Greece; 7 Department of Environmental Chemistry, Institute of Environmental Assessment and Water Research, Barcelona, Spain; 8 Institute for Risk Assessment Sciences, Division for Environmental Epidemiology, Utrecht University, Utrecht, the Netherlands; 9 CanSyn Chem. Corp., Toronto, Ontario, Canada; 10 Grup de Mutagènesi, Departament de Genètica i de Microbiologia, Edifici Cn, Universitat Autònoma de Barcelona, Bellaterra, Cerdanyola del Vallès, Spain

**Keywords:** bromination, bromine, chlorination, chlorine, DBPs, disinfection by-products, mutagenicity, swimming pools, Salmonella, water

## Abstract

**Background:**

Swimming pool disinfectants and disinfection by-products (DBPs) have been linked to human health effects, including asthma and bladder cancer, but no studies have provided a comprehensive identification of DBPs in the water and related that to mutagenicity.

**Objectives:**

We performed a comprehensive identification of DBPs and disinfectant species in waters from public swimming pools in Barcelona, Catalonia, Spain, that disinfect with either chlorine or bromine and we determined the mutagenicity of the waters to compare with the analytical results.

**Methods:**

We used gas chromatography/mass spectrometry (GC/MS) to measure trihalomethanes in water, GC with electron capture detection for air, low- and high-resolution GC/MS to comprehensively identify DBPs, photometry to measure disinfectant species (free chlorine, monochloroamine, dichloramine, and trichloramine) in the waters, and an ion chromatography method to measure trichloramine in air. We assessed mutagenicity with the *Salmonella* mutagenicity assay.

**Results:**

We identified > 100 DBPs, including many nitrogen-containing DBPs that were likely formed from nitrogen-containing precursors from human inputs, such as urine, sweat, and skin cells. Many DBPs were new and have not been reported previously in either swimming pool or drinking waters. Bromoform levels were greater in brominated than in chlorinated pool waters, but we also identified many brominated DBPs in the chlorinated waters. The pool waters were mutagenic at levels similar to that of drinking water (~ 1,200 revertants/L-equivalents in strain TA100–S9 mix).

**Conclusions:**

This study identified many new DBPs not identified previously in swimming pool or drinking water and found that swimming pool waters are as mutagenic as typical drinking waters.

Disinfection by-products (DBPs) represent a ubiquitous exposure in developed countries. DBPs are formed by the reaction of disinfectants (e.g., chlorine, chloramines, ozone, or chlorine dioxide) with natural organic matter and/or bromide/iodide, and they are an unintended consequence of trying to kill pathogens in drinking water and swimming pools. More than 600 DBPs have been identified in drinking water, and many of them are mutagenic or carcinogenic (Richardson 1998; Richardson et al. 2007). This complex mixture of DBPs includes volatile and skin-permeable DBPs, such as trihalomethanes (THMs) and haloketones (Erdinger et al. 2004: Xu and Weisel 2004, 2005). Inhalation and dermal absorption, which are the primary routes of exposure to DBPs during swimming, leads to higher blood levels of THMs than do oral exposures (Ashley et al. 2005; Haddad et al. 2006; Leavens et al. 2007).

Swimming pools constitute environments with high levels of DBPs in water and air due to continuous disinfection and constant organic load from bathers (e.g., urine, sweat, cosmetics, skin cells, and hair) (Kim et al. 2002; LaKind et al. 2010). One of the most prevalent DBPs in chlorinated swimming pools is THMs (Aggazzotti and Predieri 1986; Beech et al. 1980; Judd and Jeffrey 1995), with average concentrations ranging from 16 μg/L (Santa Marina et al. 2009) to 132 μg/L (Chu and Nieuwenhuijsen 2002). Given the high nitrogen content of organic matter from bathers, nitrogenated species such as haloacetonitriles, nitrosamines, and chloramines are found in swimming pool water (Héry et al. 1995; Kim et al. 2002; Walse and Mitch 2008; Zwiener et al. 2007).

Chronic exposure to DBPs through different routes has been associated with an increased risk for bladder cancer (International Agency for Research on Cancer 2004; Villanueva et al. 2004, 2007). Trichloramine and other volatile chemicals in swimming pools are respiratory irritants; pool attendance has been associated with asthma and other respiratory effects in Olympic swimmers and pool workers, and less clearly with recreational adult swimmers and children (Goodman and Hays 2008; Jacobs et al. 2007; Stav and Stav 2005; Weisel et al. 2009). However, the mechanisms are poorly understood, and it is not known with certainty whether trichloramine or other volatile pool DBPs are responsible.

Despite the public health relevance, only a few studies, most rather recent, have investigated the chemistry and potential health effects of swimming pool water (Weisel et al. 2009; Zwiener et al. 2007). A complete chemical characterization of DBPs in indoor swimming pools has not been reported. The only mutagenicity study of swimming pool water reported that organic extracts from three public indoor pools in Victoria, British Columbia (Canada), were mutagenic in *Salmonella* TA100 (Honer et al. 1980). The authors found that acidified extracts eluted with ether were more mutagenic in the presence of metabolic activation (rat liver S9) than without S9; however, nonacidified extracts eluted with acetone were mutagenic only in the absence of S9. One genotoxicity study of swimming pool water reported that the water and its fractions induced DNA damage in Hep-G2 cells (comet assay) and that most of the genotoxicity was in the lower-molecular-weight DBP fraction (Glauner et al. 2005). Another study using the comet assay showed that pool water was more genotoxic than the source tap water and that the type of disinfectant and illumination conditions altered the genotoxicity (Liviac et al. 2010).

The present study involves an investigation in Barcelona, Spain, where we examined 49 healthy nonsmoking volunteers before and after swimming in public swimming pools treated with either chlorine or bromine to evaluate personal exposure and a range of biomarkers of genotoxicity and respiratory damage (Font-Ribera et al. 2010; Kogevinas et al. 2010). To complement the exposure assessment, we evaluated the mutagenicity of the pool waters in the *Salmonella* mutagenicity assay and screened for DBPs, comprehensively identifying most DBPs detected and quantifying a few targeted DBPs and disinfectant species (THMs, chlorine, monochloramine, dichloramine, and trichloramine) in the pool waters and in the air phase above the water (THMs and trichloramine). In this article we present a comprehensive identification of DBPs and disinfectant species in the pool waters and compare the species formed in chlorinated versus brominated pool water with the corresponding mutagenicity of the waters.

## Materials and Methods

### Sampling

Water samples were collected from two large public swimming pools in Barcelona, Spain. One pool (33 × 25 × 2 m in size) used chlorine (sodium hypochlorite) for disinfection, after sand filtration; the other pool (20.9 × 13.2 × 1.3 m in size) used bromine (1-bromo-3-chloro-5,5-dimethyl-2,4-imidazolidinedione) for disinfection, after sand and granulated carbon filtration. Floor-to-ceiling height was 10 m and 5 m in the chlorinated and brominated pools, respectively.

### Quantitative analyses

Free chlorine, monochloramine, dichloramine, trichloramine, and THMs were measured in composite pool water samples (1 L) collected from four different locations. Free chlorine, monochloramine, dichloramine, and trichloramine were measured immediately using the *N*,*N*-diethyl-*p*-phenylenediamine (DPD) method with a portable photometer (DINKO Instruments, Barcelona, Spain). Water samples (40 mL) for THM measurements were quenched with 5 mg sodium thiosulfate and stored at 4°C until analysis on the same day. Chloroform, bromodichloromethane, dibromochloromethane, and bromoform were measured using purge-and-trap gas chromatography/mass spectrometry (GC/MS) (Tekmar 3100, Voyager MS; ThermoFisher, Waltham, MA, USA) following the method described by Lourencetti et al. (2010). Sixty-eight samples were collected from the chlorinated pool and 12 from the brominated pool for these quantitative analyses.

Indoor air samples to measure THMs were collected with a pump located 60 cm above the floor and 1.5 m from the pool border. Air was pumped (7 mL/min) for 20 min through a Tenax TA cartridge (1.8 g; Supelco, Sigma-Aldrich, St. Louis, MO, USA). Quality control was assured by daily calibration of the pump. Chloroform, bromodichloromethane, dibromochloromethane, and bromoform were determined through an automatic thermal desorption unit (ATD 400; Perkin-Elmer, Madrid, Spain) coupled to a GC-electron capture detector (Perkin-Elmer). Sixty-eight air samples were collected from the chlorinated pool, and 12 from the brominated pool.

Trichloramine was measured in pool air samples by pumping air (1.2 L/min) for 115 min, within 1 m from the water and at a height of 60 cm from the floor level, using a method described originally by Héry et al. (1995). Trichloramine was captured on two 37-mm quartz-fiber filters, one of which was placed as a backup filter, both impregnated with 500 mL of a solution of diarsenic trioxide (4 g/L As_2_O_3_), sodium carbonate (40 g/L Na_2_CO_3_), and glycerol (40 mL/L C_3_H_8_O_3_). These filters were placed in a sampling cassette with a 37-mm cellulose support filter and a 37-mm Teflon filter to prevent chloride from airborne water droplets from being captured in the sampler. Impregnated filters were desorbed in 10-mL ultra-high quality, ultrapure water (specific conductivity, 17.8 MΩ/cm at 25°C), sonicated for 30 min, and centrifuged for 15 min at 3,000 × *g* after sampling. Trichloramine was reduced to chloride and subsequently analyzed by ion chromatography (Dionex DX100; Dionex BV, Bavel, the Netherlands; AS14A guard and AS14 highly selective anion column with self-regenerating suppressor; conductivity detector; flow rate, 1.0 mL/min). Six samples were collected from the chlorinated pool, and three from the brominated pool.

### Preparation of water extracts and concentrates

For comprehensive GC/MS analyses and mutagenicity testing, pool water samples were collected at approximately noon on five different sampling events for the chlorinated pool (7 and 24 May, 11 June, and 17 September 2007) and two different sampling events for the brominated pool (16 July and 15 October 2007). Samples (28 L each) were collected using 2-L Teflon bottles (headspace-free) and were shipped overnight in coolers with icepacks to the U.S. Environmental Protection Agency laboratory in Athens, Georgia (USA). Water samples were extracted immediately upon arrival using the XAD resin process of Richardson et al. (2008) [for further details, see Supplemental Material (doi:10.1289/ehp.1001965)]. The final extract was divided for comprehensive GC/MS analysis (1.0 mL, equivalent to 20 L water) and mutagenicity analysis (0.4 mL, equivalent to 8 L water, or 20,000×).

### Comprehensive GC/MS analyses

Half of the 1.0-mL extract was derivatized with diazomethane [see Supplemental Material (doi:10.1289/ehp.1001965)] to enable the identification of haloacids (through their corresponding methyl esters); the other half was analyzed directly for other DBPs.

Comprehensive GC/MS analyses were performed on a high-resolution magnetic sector mass spectrometer (Autospec; Waters, Inc., Milford, MA, USA) equipped with an Agilent model 6890 gas chromatograph (Agilent, Santa Clara, CA, USA) and operated at an accelerating voltage of 8 kV and source temperature of 200°C, in both low-resolution (1,000) and high-resolution (10,000) modes. Injections of 1 μL extract were introduced via a split/splitless injector (in splitless mode) onto a GC column (DB-5, 30-m × 0.25-mm i.d. 0.25-μm film thickness; J&W Scientific/Agilent, Santa Clara, CA, USA). The GC temperature program consisted of an initial temperature of 35°C (4 min) and an increase at 9°C/min to 285°C (held for 30 min). Transfer lines were held at 280°C, and the injection port at 250°C.

For qualitative identifications, mass spectra of unknown compounds in the finished and raw water concentrates were subjected initially to library database searching (using the 2005 NIST Mass Spectral Library database; National Institute of Standards and Technology, Gaithersburg, MD). However, many DBPs were not present in the library database; in those cases, and also where a library match was insufficient to offer a tentative identification, high-resolution MS was used to provide empirical formulas for molecular ions and fragments. Mass spectra were also interpreted extensively to provide tentative structural identifications. When possible, pure standards were obtained to confirm identifications through a match of GC retention times and mass spectra.

### Chemical standards

Chemical DBP standards were either synthesized (CanSyn Chem. Corp., Toronto, ON, Canada) or purchased at the highest level of purity (Sigma-Aldrich, Milwaukee, WI, USA). The synthesis of (*E*)- and (*Z*)-bromochlorobutenedioic acid is presented in Supplemental Material (doi:10.1289/ehp.1001965).

### Mutagenicity assays

The 20,000× XAD/ethyl acetate extracts described above were solvent-exchanged into dimethyl sulfoxide (DMSO; Burdick and Jackson, Muskegon, MI, USA) and diluted to 10,000× and 1,000×. We performed the standard plate-incorporation *Salmonella* (Ames) mutagenicity assay (Maron and Ames 1983) in the base-substitution strain TA100 (*hisG46 rfa* Δ*uvrB*, pKM101), obtained from B.N. Ames, Children’s Hospital Oakland Research Institute (Oakland, CA, USA). We also tested the extracts in *Salmonella* strain RSJ100, which expresses the rat *GSTT1* gene, and its control strain TPT100. These strains are homologous to TA100 except that they do not contain the pKM101 plasmid and either do or do not express *GSTT1* (Thier et al. 1993). These strains were obtained from F.P. Guengerich (Vanderbilt University, Nashville, TN, USA). We did not use S9 mix because we assumed that pool water was similar to drinking water, and drinking water extracts are most mutagenic in the absence of S9 mix (Takanashi et al. 2009).

Extracts were tested up to 100 μL/plate over a dose range of 0.01–0.3 L-equivalents (L-eq)/plate based on doses used for drinking water (DeMarini et al. 1995) and a dose-range–finding study. Because of limited amounts of samples available for testing in all three bacterial strains, only two samples from the chlorinated pool (C4 and C5) and two from the brominated pool (B1 and B2) were evaluated for mutagenicity, each at one plate per dose in single experiments. We incubated the plates for 3 days at 37°C, counted the colonies [revertants (rev)] on an automatic colony counter, and calculated linear regressions over the linear portion of the dose–response curves to determine the mutagenic potencies (rev/L-eq). We defined a positive result as a dose-related response with two or more times the number of revertants observed in the DMSO control. The positive control for all strains was sodium azide at 3 μg/plate.

We calculated linear regressions, slope values, the standard error of the slopes, and *r*^2^ values of the dose–response curves and then compared the regression lines between strains RSJ100 (*GST+*) and TPT100 (*GST−*) to obtain *p*-values using Statgraphics Centurion XVI (Statpoint Technologies, Inc., Warrenton, VA, USA). The model tests the null hypothesis that the slopes are equal; we set α = 0.05 for the *F*-test with 2 degrees of freedom.

## Results

### DBPs

Table 1 lists levels of free chlorine, chloramines, and THM species in the pool water and air. Although we did not detect trichloramine in the pool waters, we did find mean levels of 0.29 and 0.08 mg/m^3^ in the chlorinated and brominated pool air, respectively, indicating that most of it volatilized from the water into the air (Font-Ribera et al. 2010). We identified > 100 DBPs comprehensively in the pool waters [Table 2; see also Supplemental Material, Figure S1 (doi:10.1289/ehp.1001965)], including a large number of haloacids, halomethanes, haloacetonitriles, haloaldehydes, haloketones, halonitromethanes, haloamides, haloalcohols, and halophenols. All of these contained either bromine or chlorine; we detected no iodinated DBPs. Most DBPs have not been reported previously for swimming pool waters, and many were not present in the mass spectral library database.

**Figure 1:**
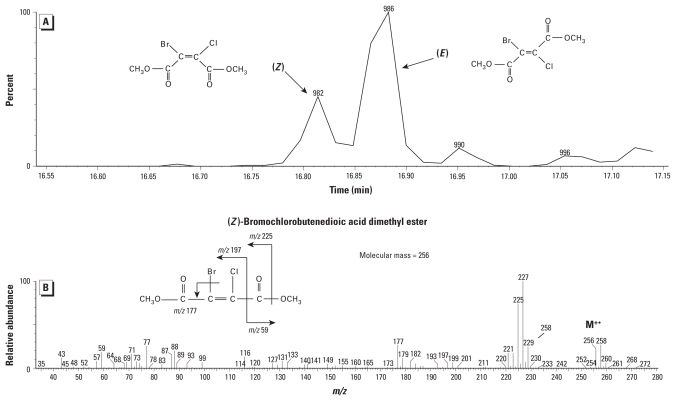
(*A*) GC/MS chromatogram showing (*Z*)- and (*E*)-2-bromo-3-chlorobutenedioic acid dimethyl ester isomers. (*B*) Electron ionization mass spectrum for (*Z*)-2-bromo-3-chlorobutenedioic acid dimethyl ester.

The identification of (*E*)- and (*Z*)-bromochlorobutenedioic acid (in their corresponding methyl ester forms) illustrates how we identified unknown DBPs. They eluted at different retention times (Figure 1A) but exhibited similar mass spectra (indicative of isomeric structures), each containing *m*/*z* 256/258/260, 225/227/229, and 59 (Figure 1B). The loss of 31 Da (typically OCH_3_) at *m*/*z* 225 and the presence of *m*/*z* 59 [typically C(O)OCH_3_] suggested the presence of a carboxylic acid methyl ester in the structures, with a molecular ion of *m*/*z* 256/258/260. Further, the *m*/*z* 256/258/260 isotopic pattern was indicative of one bromine and one chlorine atom, matching the calculated theoretical pattern [see Supplemental Material, Figure S2 (doi:10.1289/ehp.1001965)]. This pattern results from the overlap of the two natural isotopes of bromine (^79^Br and ^81^Br) with the two natural isotopes of chlorine (^35^Cl and ^37^Cl). This information suggested a tentative structural identification of bromochlorobutenedioic acid dimethyl ester, with a monoisotopic molecular mass of 256 Da. Exact mass data provided by high-resolution MS supported this empirical formula (C_6_H_6_O_4_ClBr). The observed exact mass of the stronger molecular ion isotopic peak (*m*/*z* 257.9116) was within 0.0002 Da of the theoretical mass (*m*/*z* 257.9118). This supported the general structure of bromochlorobutenedioic acid dimethyl ester; however, the exact isomer assignments could not be made by MS data alone because the spectra were too similar, which is often the case for isomers. Two structural isomers are possible for this empirical formula, (*Z*) and (*E*), representing *cis* and *trans* isomers, respectively (Figure 1A).

Fortunately, we observed both compounds in most of the pool water concentrates, so it just remained to be determined which specific isomer represented each GC/MS chromatographic peak. To make this determination, we synthesized the two possible isomers [see Supplemental Material (doi:10.1289/ehp.1001965)] and confirmed by a match of the GC retention time and mass spectra that the (*Z*) isomer is the first peak at 16.8 min and the (*E*) isomer is the second peak at 16.9 min in the pool water extracts (Figure 1A).

### Mutagenicity

**Figure 2:**
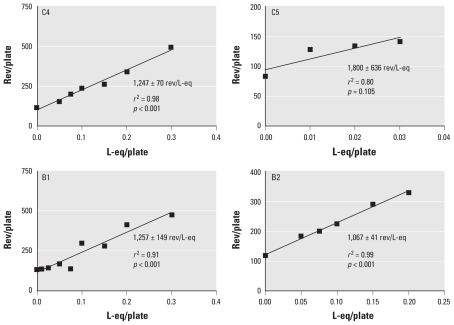
Mutagenicity in *Salmonella* TA100–S9 of two samples each from the chlorinated (C4, C5) and (B1, B2) brominated pools. Data in each curve are from Table 3 and represent a single experiment performed with one plate per dose. Slope (mutagenic potency) is rev/L-eq ± SE of the slope.

Table 3 and Figure 2 show the mutagenicity data for two samples from the chlorinated pool and two from the brominated pool in strain TA100. All of the samples were mutagenic in strain TA100 except for sample C5, which was the only sample that showed toxicity—based on a reduction of rev/plate in TA100 at the highest doses (0.04 and 0.05 L-eq/plate). Table 4 shows the slopes, *r*^2^ values, and standard errors of the slopes for these data; the average mutagenic potency of the three mutagenic samples was 1,190 rev/L-eq. Only sample B1 was significantly more mutagenic in the *GSTT1*-expressing strain relative to the nonexpressing strain (Tables 3 and 4). This indicates that some portion of the mutagenic activity of sample B1 in strain RSJ100 was due to the presence of DBPs that were activated by *GSTT1*, such as the brominated THMs (DeMarini et al. 1997; Pegram et al. 1997).

## Discussion

Most analytical studies of pool water have measured only a few targeted DBPs, primarily chloroform and other THMs. Consequently, this study expands considerably our knowledge of the chemical composition and mutagenicity of swimming pool water beyond the chemical analysis of two outdoor pools by Zwiener et al. (2007) and the studies on pool water mutagenicity (Honer et al. 1980) and genotoxicity (Glauner et al. 2005; Liviac et al. 2010). We found a greater number of DBPs in the chlorinated and brominated indoor pools studied here than have been found in chlorinated outdoor pools (Zwiener et al. 2007), which was not surprising, considering that DBPs can be volatilized or photolyzed (Lekkas and Nikolaou 2004) in outdoor settings. In addition, although most people assume that chlorine levels in swimming pools are much higher than in chlorinated drinking water, the mean level of free chlorine (1.28 and 0.50 mg/L in the chlorinated and brominated pools, respectively) was similar to that found typically in drinking water.

Because little is known regarding the mutagenicity and DBP composition of swimming pool water, we compared our data with those for drinking water, which are much more extensive (Richardson et al. 2007). In addition, the pool water composition and mutagenicity reported here can be used to better understand the reported health effects of swimming pool water, such as asthma, irritation of eyes/throat/skin, and bladder cancer (Weisel et al. 2009; Zwiener et al. 2007).

### Nitrogen-containing DBPs

In general, we observed more nitrogen-containing DBPs (N-DBPs) in these pool water samples than are found typically in chlorinated drinking water. For example, we found a greater number of haloamides, halonitriles, haloanilines, haloanisoles, and halonitro-compounds than typically found in drinking water, and several chemicals within these families have not been reported previously in drinking water. In addition, we detected mono- and dichloramine in the pool waters (means of 0.29 and 0.38 mg/L, respectively, for mono- and dichloramine in the chlorinated pool, and a mean of 0.27 mg/L for monochloramine in the brominated pool). Because DPD analysis of chloramines cannot differentiate organic from inorganic forms of these compounds, it is possible that these levels are overestimated by the occurrence of organic chloramines in the swimming pool waters. Model studies with batch experiments show that survival of chloramines depends on the chlorine:nitrogen ratio (Jafvert and Valentine 1992). Considering this, the low concentrations of mono- and dichloramine reported in Table 1 are consistent with chlorine oxidation of continuous supplies of small amounts of nitrogen compounds coming from urine, sweat, skin, and other human residues. These levels are also similar to those reported by other researchers who used membrane-introduction MS, which does not have issues with interferences from organic chloramines (Shang and Blatchley 1999; Weaver et al. 2009).

The N-DBPs, including chloramines, were not surprising to find because pool waters have a greater contribution of nitrogen-containing precursors due to human inputs. Because chloramines are known to cause eye irritation and other problems, pool operators generally try to add enough chlorine to get beyond the “break point,” such that these chloramines are destroyed, leaving residual chlorine (Ford 2007; World Health Organization 2000). However, the amount of chlorine needed to reach “break point” is also dependent on other amines in the water. As we observed in this study, this goal is not always achieved because of continuous human inputs and rapid reactions forming chloramines. A few other N-DBPs also have been reported in swimming pool waters, including organic chloramines (Li and Blatchley 2007), and nitrosamines (Walse and Mitch 2008), several of which are carcinogenic.

### Comparison of brominated versus chlorinated pool waters

Bromoform levels were much higher in the pools treated with bromine versus chlorine, but interestingly, other DBPs and their levels were similar in brominated versus chlorinated pools, likely owing to the high levels of bromide present already in Barcelona source waters (Ventura and Rivera 1985) that feed into drinking water treatment and further swimming pool treatment (Judd and Jeffrey 1995). In addition, when THMs are compared on a molar basis, the chlorinated pool actually contained somewhat higher levels of total THMs (mean, 306 nM) than the brominated pool (mean, 242 nM); this was possibly due to the carbon filtration used at the brominated pool that was not used at the chlorinated pool.

### Mutagenicity

In the only other mutagenicity study of swimming pool water, Honer et al. (1980) found that three public indoor pools in Victoria, British Columbia (Canada), produced approximately 20,000 rev/L-eq in *Salmonella* TA100 (without S9 mix), compared with our finding of an average of 1,190 rev/L-eq in the two indoor pools in Barcelona. However, their solvent-extraction method was considerably different from ours, involving ether and acetone, whereas we used ethyl acetate. Although a direct comparison of the data is not possible, our study confirms their pioneering work from three decades ago showing that swimming pool water is mutagenic.

In general, extracts of drinking water induce an average of 1,100 rev/L-eq in *Salmonella* strain TA100 (without S9 mix)(Takanashi et al. 2009); however, values as high as approximately 15,000 rev/L-eq have been reported (Egorov et al. 2003). Concentration methods such as reverse osmosis recover levels of mutagenic activity lower than those recovered by XAD resin (Claxton et al. 2008), which is why we used XAD to prepare extracts of pool water. Our finding that the pool water mutagenicity was similar to that of drinking water may reflect the fact that the levels of mutagenic DBPs in the pool waters were similar to those in drinking water, despite the differences in the levels of specific classes of DBPs described above in pool versus drinking water.

Our finding that some of the mutagenic activity of one sample (B1) from the brominated pool water was due to activation by GSTT1 suggests the presence of compounds that are activated to mutagens by this enzyme, such as brominated THMs (DeMarini et al. 1997; Pegram et al. 1997), some methylene dihalides and bifunctional butanes (Thier et al. 1995), and/or 1,1-dichloropropene (Granville et al. 2005). Our chemical analysis (Table 1) showed that sample B1 had high concentrations of brominated THMs, especially bromoform. The high cytotoxicity and lack of mutagenicity of sample C5 may reflect the fact that the concentration of chloroform was 30% higher in this sample than in sample C4. Perhaps the higher concentration of chloroform, which is cytotoxic but not mutagenic, produced the observed cytotoxicity, preventing detection of mutagenic activity of the other DBPs present in sample C5. As reviewed by Richardson et al. (2007), many other DBPs in drinking water that we have now identified in pool waters are known to be mutagenic and/or carcinogenic, including the haloacetic acids, halonitromethanes, haloamides, haloacetonitriles, and unregulated haloacids (Plewa et al. 2008a, 2008b; Richardson et al. 2007).

In addition to the mix of mutagenic DBPs identified in the pool water, many other DBPs have not yet been studied for health effects, and no doubt, many other DBPs remain to be identified that also may contribute to the observed mutagenicity of swimming pool water. In this regard, the study by Glauner et al. (2005), which found that the low-molecular-weight fraction of extracts from indoor and outdoor pools in Germany was the most potent of all fractions for inducing DNA damage in mammalian cells (using the comet assay), suggests that the low-molecular-weight DBPs may be most responsible for the genotoxic effect of swimming pool water. Also using the comet assay, Liviac et al. (2010) found that pool water was more genotoxic than the source tap water; a similar analysis using the Ames assay would help in characterizing the relative mutagenicity of drinking versus pool water. Sources of mutagens unique to pool water could include active agents in sunscreens, which can be transformed to mutagens by exposure to free chlorine under conditions similar to swimming pool water (Nakajima et al. 2009).

Our limited data indicate that the mutagenic potencies of chlorinated versus brominated pool waters were similar, as were the dose ranges over which the pool waters were mutagenic, approximately 0.1–0.3 L-eq/plate (Figure 2). In contrast, the typical dose range for drinking water mutagenicity is 0.3–1.5 L-eq/plate (DeMarini et al. 1995). This difference reflects the considerably higher toxicity of swimming pool water relative to drinking water, with the highest testable mutagenic dose of pool water being the lowest mutagenic dose of drinking water.

## Conclusions

We identified > 100 DBPs in two indoor pools, including a prevalence of N-DBPs, likely formed from nitrogen-containing precursors from human inputs. This study provides the most comprehensive analysis to date of DBPs in swimming pool waters, as well as a clear demonstration of their mutagenicity. In addition, many DBPs we identified are new and have not been reported previously in pool waters. Bromoform levels were much greater in the brominated versus chlorinated pools. Compared with previous research on outdoor pools, we found a much greater number of DBPs in these indoor pools.

The mutagenicity of these pool waters was similar to that of drinking water, indicating that the levels of mutagenic DBPs are similar in both waters. Subjects who swam in the mutagenic, chlorinated pool water evaluated in this study had increases in genotoxicity biomarkers that were associated with the concentrations of brominated THMs, but not chloroform, in their exhaled breath (Kogevinas et al. 2010). These findings are especially relevant with regard to a case–control study by Cantor et al. (2010) in this issue that identifies an enhanced risk for bladder cancer associated with DBP exposure among people with genotypes that metabolize various DBPs. Further research on a wide array of swimming pools under various conditions of maintenance and use are warranted based on the limited but developing data now available on the chemical composition and health risks of swimming pool water.

## Figures and Tables

**Table 1:** Free chlorine, chloramine, and THM levels in the swimming pools.

	Chlorinated pool				Brominated pool			
Chemical and concentration	Mean ± SD	Minimum	Maximum	*n*	Mean ± SD	Minimum	Maximum	*n*
Water								
Free chlorine (mg/L)	1.28 ± 0.43	0.52	2.35	68	0.50 ± 0.16	0.32	0.7	4
Monochloramine (NH_2_Cl) (mg/L)	0.29 ± 0.11	0.10	0.64	68	0.27 ± 0.03	0.24	0.3	4
Dichloramine (NHCl_2_) (mg/L)	0.38 ± 0.14	< 0.01	0.65	68	< 0.01	< 0.01	< 0.01	4
Trichloramine (NCl_3_) (mg/L)	< 0.10	< 0.10	< 0.10	68	< 0.10	< 0.10	< 0.10	4
Chloroform (CHCl_3_) (μg/L)	15.4 ± 3.5	8.4	20.8	68	0.2 ± 0.1	0.1	0.3	12
Bromodichloromethane (CHCl_2_Br) (μg/L)	14.2 ± 4.2	9.3	26.8	68	0.4 ± 0.2	0.2	0.7	12
Dibromochloromethane (CHClBr_2_) (μg/L)	12.8 ± 4.4	6.5	22.6	68	2.4 ± 0.2	2.1	2.7	12
Bromoform (CHBr_3_) (μg/L)	7.2 ± 3.2	3.0	16.5	68	57.2 ± 4.4	52.0	64.3	12
Total THMs (μg/L)	49.6 ± 10.6	35.2	75.2	68	60.2 ± 4.7	54.4	67.2	12

Air								
Trichloramine (NCl_3_) (mg/m^3^)	0.29 ± 0.10	0.17	0.43	6	0.08 ± 0.01	0.07	0.10	3
Chloroform (CHCl_3_) (μg/m^3^)	32.1 ± 11.9	11.9	61.6	68	4.4 ± 2.3	1.7	9.4	12
Bromodichloromethane (CHCl_2_Br) (μg/m^3^)	14.9 ± 4.5	7.5	23.4	68	2.9 ± 1.0	1.7	4.8	12
Dibromochloromethane (CHClBr_2_) (μg/m^3^)	14.0 ± 4.2	6.1	26.2	68	7.3 ± 1.3	6.1	9.7	12
Bromoform (CHBr_3_) (μg/m^3^)	11.0 ± 4.6	4.4	22.6	68	74.9 ± 17.6	53.3	101.4	12
Total THMs (μg/m^3^)	72.1 ± 20.7	44.0	124.9	68	89.5 ± 21.9	63.1	124.7	12

**Table 2:** DBPs identified in pool waters.

	Sample						
DBP	C1	C2	C3	C4	C5	B1	B2
Haloalkanes							
*Chloroform*^a^	x	x	x	x	x	x	x
*Bromodichloromethane*	x	x	x	x	x	x	x
*Dibromochloromethane*	x	x	x	x	x	x	x
*Bromoform*	x	x	x	x	x	x	x
Dibromomethane	x	x	x	x	x	x	x
Bromotrichloromethane							
Dibromodichloromethane					x		
1,1,2-Trichloroethane					x		x

Haloacetic acids							
*Chloroacetic acid*	x		x	x	x		
*Bromoacetic acid*			x	x	x	x	x
*Dichloroacetic acid*	x	x	x	x	x		
*Bromochloroacetic acid*	x	x	x	x	x	x	x
*Dibromoacetic acid*	x	x	x	x	x	x	
*Trichloroacetic acid*	x	x	x	x	x	x	x
*Bromodichloroacetic acid*	x	x	x	x	x	x	x
*Dibromochloroacetic acid*	x	x	x	x	x	x	x
*Tribromoacetic acid*	x	x	x	x	x	x	x

Other haloacids							
3-Bromopropenoic acid	x						
*2,2-Dichloropropanoic acid*	x	x	x	x	x		
*3,3-Dichloropropenoic acid*	x	x	x	x	x		
cis*-2,3-Bromochloropropenoic acid*	x	x	x		x	x	x
trans*-2,3-Bromochloropropenoic acid*	x	x	x		x	x	x
*2,3-Dibromopropanoic acid*	x		x		x	x	x
cis*-2,3-Dibromopropenoic acid*			x	x	x		x
*trans*-2,3-Dibromopropenoic acid				x	x		x
3,3-Dibromopropenoic acid				x	x	x	x
*Trichloropropenoic acid*	x	x	x	x	x	x	x
*2-Bromo-3,3-dichloropropenoic acid*	x	x	x	x	x	x	x
*(*E*)-3-Bromo-2,3-dichloropropenoic acid*	x	x	x	x	x	x	x
*(*Z*)-3-Bromo-2,3-dichloropropenoic acid*	x	x	x	x	x	x	x
2,2-Dichlorobutanoic acid	x	x	x	x			
*cis*-Bromobutenoic acid			x	x	x	x	x
*trans*-Bromobutenoic acid				x		x	x
2,2-Dichlorobutenoic acid					x		
2,3-Dibromobutenoic acid						x	x
*2-Chloro-3-methylbutanoic acid*	x	x	x	x	x		
Chlorophenylacetic acid			x		x		
3,5-Dibromobenzoic acid							x
*Tribromopropenoic acid*						x	

Halodiacids							
cis*-Bromobutenedioic acid*	x	x	x	x	x	x	x
trans*-Bromobutenedioic acid*			x	x	x	x	x
*cis-*Dichlorobutenedioic acid			x	x		x	
*trans*-Dichlorobutenedioic acid			x	x			
cis*-Bromochlorobutenedioic acid*	x	x	x	x	x		x
trans*-Bromochlorobutenedioic acid*	x	x	x	x	x	x	x
cis*-Dibromobutenedioic acid*	x	x	x	x	x	x	x
*(*E*)-2-Chloro-3-methylbutenedioic acid*	x		x				
(*E*)-2-Bromo-3-methylbutenedioic acid						x	

Haloaldehydes							
*Dichloroacetaldehyde*	x	x					
*Bromochloroacetaldehyde*			x	x	x		
*Dibromoacetaldehyde*			x	x	x	x	x
*Trichloroacetaldehyde* (*chloral hydrate*)	x	x	x	x	x		
*Bromodichloroacetaldehyde*	x	x	x	x			
*Dibromochloroacetaldehyde*	x	x	x	x	x		
*Tribromoacetaldehyde*	x	x	x	x	x		
3-Bromo-4-methoxybenzaldehyde		x	x	x	x	x	x

Halonitriles							
*Bromoacetonitrile*				x		x	
*Dichloroacetonitrile*	x	x	x	x	x		
*Bromochloroacetonitrile*	x	x	x	x	x	x	
*Dibromoacetonitrile*	x	x	x	x	x		
*Trichloroacetonitrile*	x						

Haloketones							
*Bromopropanone*						x	x
1,1-Dichloropropanone		x	x				
*1-Bromo-1-chloropropanone*			x		x		
*1,1-Dibromopropanone*						x	x
1,3-Dibromopropanone						x	x
*1,1,1-Trichloropropanone*	x	x	x	x	x		
*1,1,3-Trichloropropanone*		x	x	x	x		
1-Bromo-1,1-dichloropropanone		x		x	x		
1,1,1-Tribromopropanone			x	x	x		
*1,1,3,3-Tetrachloropropanone*	x	x	x	x	x		
1,1-Dibromo-3,3-dichloropropanone							
Pentachloropropanone				x	x		
Dichlorofurandione				x	x		
1-Chloro-2-butanone				x	x		
1-Bromo-2-butanone			x			x	
Tetrachlorohydroquinone			x	x	x		

Halonitromethanes							
*Dibromonitromethane*			x	x	x	x	x

Haloamides							
*Dichloroacetamide*	x	x	x				
*Bromochloroacetamide*	x	x					
*Dibromoacetamide*	x	x	x	x	x	x	x
*Bromodichloroacetamide*	x						
*Dibromochloroacetamide*	x	x	x				
*Tribromoacetamide*			x				

Haloalcohols							
2,2,2-Trichloroethanol			x				
1,1,1-Trichloropropanol	x		x	x			

Other halogenated DBPs							
3-Chlorobenzeneacetonitrile			x				
2,6-Dichloro-4-methylphenol			x	x	x		
2-Bromo-4-chlorophenol					x		
Trichlorophenol	x	x	x	x	x		
Bromodichlorophenol	x	x		x	x		
Tribromophenol	x		x			x	
2-Bromo-4-chloro-6-methylphenol		x	x	x	x		
Dibromomethylphenol		x	x				
2,4-Dibromo-1-methoxybenzene					x	x	
2,3,4-Trichlorobenzeneamine				x		x	x
Dibromochloroaniline					x		
2-Bromo-4-chloroanisole		x	x	x			
3,4,5-Tribromo-*1H*-pyrazole						x	
2,6-Dibromo-4-nitrophenol						x	
2,6-Dibromo-4-nitrobenzeneamine						x	x

Nonhalogenated DBPs/contaminants							
Propionamide	x						
*Benzaldehyde*	x	x	x	x	x	x	x
*Benzoic acid methyl ester*			x				
Benzeneacetonitrile	x					x	
*Phthalic acid*	x		x	x			
*Diethylphthalate*	x						
*Benzophenone*					x		

Samples C1–C5 represent five samples from the chlorinated pool; B1 and B2 represent two samples from the brominated pool. “X” indicates that a particular DBP was identified in that sample.

a DBPs shown in italics were confirmed through the analysis of authentic standards; all others should be considered tentative identifications.

**Table 3:** Mutagenicity of pool waters in *Salmonella*.

	Strain (rev/plate)		
Sample L-eq/plate	TPT100 (*GST*−)	RSJ100 (*GST+*)	TA100
C4			
0	16, 19	10, 14	127, 115, 111
0.05	27	28	158
0.075	39	10	200
0.1	52	60	238
0.15	44	57	264
0.2	69	81	343
0.3	87	70^a^	495

C5			
0	9, 19, 12	6, 6, 5	75, 83, 94
0.01	24	8	128
0.02	23	18	134
0.03	21^a^	27	142
0.04	21^a^	23^a^	115^a^
0.05	12^a^	25^a^	109^a^

B1			
0	27, 18, 20	9, 8, 5	130, 128
0.01	14	14	132
0.025	16	7	137
0.05	16	19	164
0.075	6	15	130
0.1	19	26	294
0.15	19	28	274
0.2	26	42	407
0.3	33	54^a^	471

B2			
0	16, 19	10, 14	127, 115, 111
0.05	19	24	182
0.075	29	28	199
0.1	30	26	225
0.15	37	34	290
0.2	32^a^	34^a^	330
0.3	26^a^	30^a^	373^a^

The average rev/plate for the positive control, sodium azide (3 μg/plate), was 910 for TPT100, 519 for RSJ100, and 645 for TA100. The average rev/plate for the solvent blank (2 L-eq/plate) was 10 for RSJ100 and 128 for TA100; it was not tested in TPT100.

a Numbers were outside of the linear range of the dose response and were not used to calculate the linear regressions for potency values (Figure 2, Table 4).

**Table 4:** Mutagenic potencies of pool water samples in GST− and GST+ strains of *Salmonella*.

	Rev/L-eq ± SE (*r*^2^)		
Sample	TPT100 (*GST*−)	RSJ100 (*GST+*)	*p*-Value
C4	228.2 ± 27 (0.93)	357.9 ± 95 (0.78)	0.131
C5	500.0 ± 346 (0.68)	730.0 ± 128 (0.94)	0.508
B1	54.8 ± 20 (0.51)	159.1 ± 22 (0.90)	0.000
B2	136.0 ± 26 (0.90)	136.0 ± 28 (0.89)	0.194
